# Excess mortality impact of two epidemics of pandemic influenza A(H1N1pdm09) virus in Hong Kong

**DOI:** 10.1111/irv.12196

**Published:** 2013-11-07

**Authors:** Peng Wu, Edward Goldstein, Lai-Ming Ho, Joseph T Wu, Thomas Tsang, Gabriel M Leung, Benjamin J Cowling

**Affiliations:** a School of Public Health, Li Ka Shing Faculty of Medicine, The University of Hong Kong, Hong Kong Special Administrative RegionChina; b Center for Communicable Disease Dynamics, Department of Epidemiology, Harvard School of Public HealthBoston, MA, USA; c Centre for Health Protection, Department of Health, Government of the Hong Kong Special Administrative Region, Hong Kong Special Administrative RegionChina

**Keywords:** Excess mortality, H1N1pdm09, impact, influenza, pandemic

## Abstract

Hong Kong experienced two large epidemics of pandemic influenza A(H1N1pdm09). We used regression methods to estimate the excess mortality associated with each epidemic. The first epidemic of H1N1pdm09 peaked in September 2009 and was associated with 2·13 [95% confidence interval (CI): −8·08, 11·82] excess all-cause deaths per 100 000 population. The second epidemic of H1N1pdm09 in early 2011 was associated with 4·72 [95% CI: −0·70, 10·50] excess deaths per 100 000 population. More than half of the estimated excess all-cause deaths were attributable to respiratory causes in each epidemic. The reasons for substantial impact in the second wave remain to be clarified.

## Introduction

The pandemic influenza A(H1N1)pdm09 virus (pH1N1 hereafter) has caused substantial morbidity and mortality during 2009–2010 far greater than the laboratory-confirmed infections^1^ while most studies documenting multiple epidemic waves following the 2009–2010 pH1N1 epidemic were based on laboratory-confirmed cases,^2^–^5^ which could substantially underestimate the true impact of influenza. Hong Kong experienced major epidemics of pH1N1 in 2009–2010 and again in 2011. We examined the patterns in excess mortality associated with pH1N1 during the two epidemics, compared with preceding and contemporaneous epidemics of seasonal influenza viruses.

## Methods

### Sources of data

Age-specific weekly deaths and mid-year population sizes from 1998 to 2011 were obtained from the Census and Statistics Department of the Hong Kong Government. Respiratory deaths were coded as 460-519 (ICD-9) or J00-J99 (ICD-10). Laboratory-confirmed pH1N1 infections in 2009–2010 were collected by the Hong Kong Hospital Authority.^6^ Weekly surveillance data on influenza-like illnesses, influenza virus and respiratory syncytial virus (RSV) activity in 1998–2011 were obtained from the Hong Kong Centre for Health Protection. Meteorological parameters were obtained from the Hong Kong Observatory.

### Statistical analysis

We applied linear regression models to investigate the association between weekly all-cause and respiratory mortality rates and influenza activity.^7^–^9^ Linear models were chosen to reflect the assumption that increases in influenza activity would lead to additive increases in mortality.^7^ The model allowed for adjustment for the activity of specific influenza virus types/subtypes and RSV, seasonal variation associated with meteorological variables and general trends over calendar time, the ICD code transition in 2001, and the impact of changes in the local pandemic alert level in April 2009 and May 2010 (Appendix).

The influenza-associated excess mortality rates were estimated as the difference in the predicted mortality rates from the fitted model in the presence and absence of influenza virus activity for a specific type or subtype over the duration of each epidemic period. Based on age-specific estimates of excess mortality, we derived the age-standardized excess mortality risk for the all-age group to allow for comparison of impact caused by different virus subtypes over the study period (Appendix). To account for autocorrelation in the residual errors, 95% confidence intervals for excess mortality rates were estimated with a bootstrap approach.^7^,^9^ All statistical analyses were conducted in r version 2.15.1.

## Results

**Figure 1:**
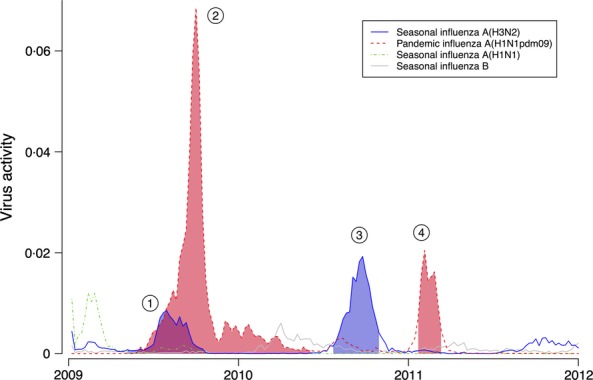
Type-/subtype-specific weekly influenza virus activity in Hong Kong from 2009 through 2011. Influenza virus activity was estimated by the weekly proportion of consultations for influenza-like illness at sentinel clinics multiplied by the weekly virus detection rate (by type/subtype) in the local public health laboratory. Shaded areas indicate the four influenza epidemics that occurred in Hong Kong from the emergence of influenza A(H1N1pdm09) virus through to the end of 2011. 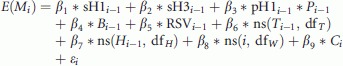 Epidemic of the seasonal influenza A(H3N2) virus in 2009 (blue area); 
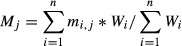
 Epidemic of influenza A(H1N1pdm09) virus epidemic in 2009–2010 (red area); 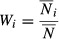 Post-pandemic epidemic of the seasonal influenza A(H3N2) virus in 2010 (blue area); and 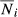 Second epidemic of influenza A(H1N1pdm09) virus epidemic in 2011 (red area).

**Figure 2:**
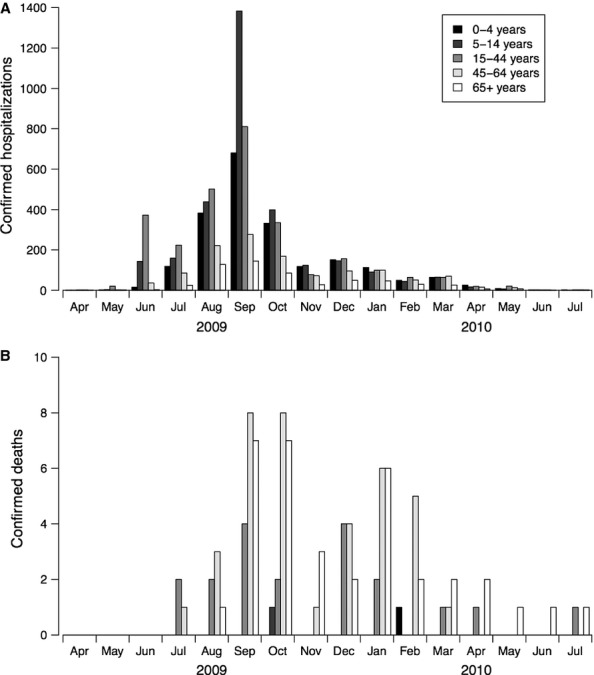
Age-specific monthly numbers of hospitalizations and deaths of patients with laboratory-confirmed influenza A(H1N1pdm09) virus infections in Hong Kong, April 2009 through July 10. (A) Hospitalizations of patients with confirmed influenza A(H1N1pdm09). (B) Deaths of patients with confirmed influenza A(H1N1pdm09). Confirmed influenza A(H1N1pdm09) virus infection was a reportable condition between April 2009 and July 2010. Data on laboratory-confirmed influenza A(H1N1pdm09) virus infections were extracted from the e-flu electronic database collated by the Hospital Authority. The changes in age pattern after the peak of the first wave of influenza A(H1N1pdm09) in October 2009 could partly be attributed to recommendations for the reduced use of laboratory testing and admission of suspected cases. Laboratory-confirmed influenza A(H1N1pdm09) hospitalizations and deaths were not available after July 2010.

In the decade prior to 2009, influenza epidemics typically occurred in Hong Kong twice per year, with peaks in activity in the winter in January–March and in the summer in June–August.^9^ This pattern was disrupted by the pandemic (Figure 1). In the summer of 2009, an epidemic of seasonal influenza A(H3N2) was followed by a larger epidemic of pH1N1 that peaked in September 2009 and did not completely fade out until mid-2010. Another epidemic of A(H3N2) occurred in the summer of 2010, and a second epidemic of pH1N1 occurred in the winter of 2011 peaking in February. There was no influenza epidemic in the summer of 2011. During the period April 2009 to July 2010, laboratory-confirmed pH1N1 virus infection was a reportable condition and there were 9647 hospitalizations and 93 deaths among patients with laboratory-confirmed pH1N1; 60 (65%) of the confirmed deaths occurred in the months April–December 2009 (Figure 2).

**Figure 3:**
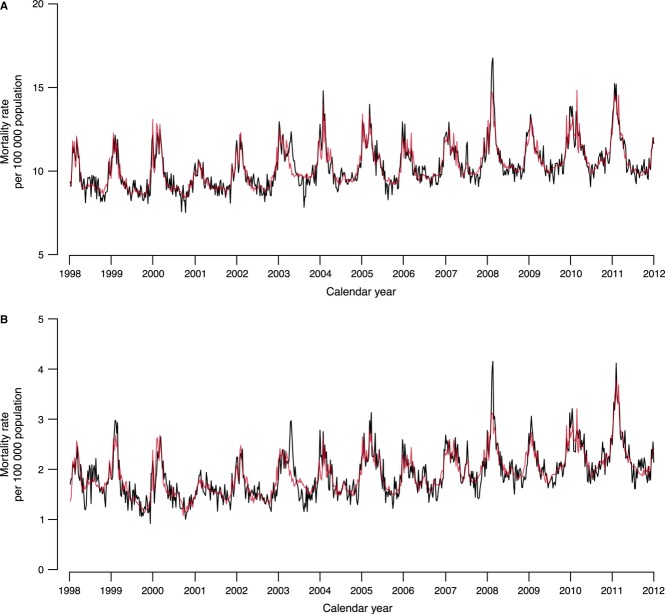
Observed (black lines) and predicted (red lines) weekly all-cause (A) and respiratory (B) mortality rates in Hong Kong from 1998 through 2011.

Our model captured the variation in all-cause and respiratory mortality in Hong Kong from 1998 through 2011 (Figure 3). In the first epidemic wave from 27 April 2009 through 24 May 2010, the excess mortality risk associated with pH1N1 was 2·13 (95% CI: −8·08, 11·82) and 1·23 (95% CI: −3·20, 5·48) per 100 000 population from all-cause and respiratory diseases, respectively (Table 1). During the second epidemic wave from 10 January through 6 March 2011, the excess mortality risk of pH1N1 was estimated to be 4·72 (95% CI: −0·70, 10·50) and 3·94 (95% CI: 1·66, 6·36) per 100 000 population for all-cause and respiratory deaths, respectively, with the majority in the elderly.

**Table 1:** Excess all-cause and respiratory mortality risks (with 95% confidence intervals), per 100 000 population associated with epidemics of seasonal influenza A(H3N2) virus and influenza A(H1N1pdm09) virus overall and by age, in Hong Kong, 2009–2011

Age	A(sH3N2) April–November 2009	A(H1N1pdm09) April 2009–May 2010	A(sH3N2) July–October 2010	A(H1N1pdm09) January–March 2011
	Risk (95% CI)	Risk (95% CI)	Risk (95% CI)	Risk (95% CI)
All-cause				
0–64	0·07 (−0·98, 1·15)	−0·23 (−3·20, 2·91)	0·11 (−0·74, 1·01)	0·43 (−1·24, 2·50)
65+	26·96 (1·80, 51·24)	18·05 (−57·29, 94·56)	46·89 (24·28, 67·73)	33·7 (−8·05, 76·87)
All ages	3·54 (0·25, 6·77)	2·13 (−8·08, 11·82)	6·15 (3·25, 8·95)	4·72 (−0·70, 10·50)
Respiratory				
0–64	0·09 (−0·25, 0·40)	0·05 (−0·77, 1·11)	0·15 (−0·15, 0·41)	0·57 (0·06, 1·15)
65+	12·19 (−0·91, 22·85)	9·18 (−24·12, 43·25)	21·21 (11·07, 30·32)	26·66 (8·66, 45·16)
All ages	1·65 (0·18, 3·00)	1·23 (−3·20, 5·48)	2·87 (1·52, 4·00)	3·94 (1·66, 6·36)

A(sH3N2), seasonal influenza A/Perth/16/2009(H3N2)-like strain; A(H1N1pdm09), pandemic influenza A/California/7/2009(H1N1)-like strain.

The seasonal influenza A(H3N2) virus which cocirculated with pH1N1 in the summer of 2009 was associated with an excess all-cause mortality risk of 3·54 (95% CI: 0·25, 6·77) per 100 000 population. In 2010, the same virus was associated with an excess all-cause mortality risk of 6·15 per 100 000 population. In both epidemics, the estimated excess deaths from all causes were approximately double the estimated excess deaths from respiratory causes (Table 1). The fitted model also permitted estimation of the impact of seasonal influenza epidemics from 1998 to 2008. The estimates were very similar to those previously reported, with the excess deaths associated with epidemics varying between 1·9 and 12·8 per 100 000 population, and seasonal A(H3N2) epidemics having greater impact than seasonal A(H1N1) and B epidemics.^9^

## Discussion

A second epidemic of pH1N1 occurred in Hong Kong in 2011 despite the first epidemic being associated with infections of up to 50% of school-age children.^6^ High incidence of infection particularly among children in Hong Kong and other countries in 2009–2010^10^,^11^ led to expectations that there would be no further large epidemics of pH1N1 unless the virus changed antigenically.^10^,^12^ Our estimate of pH1N1-associated mortality in Hong Kong was consistent with estimates from three previous studies based on deaths up to December 2009.^8^,^9^,^13^

Following the large epidemic in 2009–2010, the USA and some European countries recorded further epidemics of pH1N1 in 2010–2011 while the age distribution of reported severe cases changed compared with the first wave.^2^,^4^,^5^ Mexico reported a fourth wave in 2011–2012 season with absence of major pH1N1 epidemics in 2010 and identified a similar age shift towards elderly based on laboratory-confirmed pH1N1 hospitalizations and deaths.^3^ However, laboratory-confirmed data may not fully reflect the impact of pH1N1 epidemics.^1^ In our study, we identified a similar excess mortality impact of pH1N1 in the second wave in 2011 compared with the first wave in 2009 (Table 1), while previous studies suggested an increase in the overall severity of pH1N1 during the post-pandemic season in the UK and Germany.^14^,^15^ It is not yet clear whether age-specific severity remained the same across both epidemic waves in Hong Kong, but serologic data across both periods indicate a similar or higher cumulative incidence of infection in older adults and the elderly in the second wave compared with the first wave (Cowling BJ, unpublished data), which was likely to result from herd immunity and age-specific contact patterns of the population given that vaccination coverage was low in Hong Kong during the pandemic.^16^

The transmission dynamics underlying the possible change in age-specific impact remain unclear. It is unlikely that excess mortality in early 2011 could have been caused by other respiratory viruses. In Hong Kong, the only epidemic respiratory virus in February–March 2011 was influenza.^17^ One possible explanation for the change in age-specific excess mortality between the two waves of pH1N1 epidemic is that some form of immunity protected some adults in the first wave, but that immunity had waned before the second wave. An alternative possible explanation is that there is seasonal variation in the viability of alternative modes of transmission with environmental conditions in the winter being most supportive of aerosol transmission^18^ and because we speculate that immunity could differ for exposures via different modes.^19^ The second epidemic occurred at a similar time to winter epidemics in previous years,^9^ and environmental conditions are presumably suitable for aerosol transmission at that time of year. In contrast, in New Zealand where the first wave occurred during the usual influenza season in 2009, a second wave in 2010 had lower impact than the first wave.^20^ However, this does not explain why the incidence of laboratory-confirmed cases was low among adults in Hong Kong in early 2010 when pH1N1 continued to circulate (Figure 2). A final possible explanation is a genetic change in the virus associated with increased transmissibility in adults between the two waves, although antigenic changes have not been identified to date.

Our study has a few limitations. First, our ecologic analysis captured the overall impact of the epidemics but the regression model that we used could not account for the underlying transmission dynamics. Second, we did not have age-specific surveillance data, and our use of aggregate surveillance data on influenza and RSV activity could have led to biases in estimation of the age-specific impact of influenza for some age groups. Third, apart from RSV, we did not include laboratory information on the circulation of other respiratory viruses in the community, which might also affect influenza activity through virus interference, and could be associated with mortality rates. Fourth, the lack of statistical significance for some estimates of age-specific excess mortality does not imply that excess mortality did not occur, but may merely reflect the low number of deaths attributable to the virus in Hong Kong with a total population of approximately 7 million people. Finally, we did not examine other causes or groups of causes of death, which could provide further insights into the impact of influenza epidemics.

In conclusion, we identified the increased mortality impact of the second epidemic of pH1N1 that was similar to the impact of seasonal influenza A(H1N1) epidemics in the preceding decade.^9^ The transmission dynamics underlying a second wave of pH1N1 with substantial impact remain to be clarified.

## Appendix

### Sentinel surveillance system

The sentinel surveillance system for influenza was established in Hong Kong in 1990s. During the study period, the sentinel sites in the system included all government-sponsored public general outpatient clinics (which have below 10% of the market share for outpatient care in Hong Kong) and around 50–60 private general practitioners. The number of outpatient consultations and the proportion of consultations due to influenza-like illness were reported to the Centre for Health Protection of the Department of Health by each sentinel on a weekly basis.

Virus activity surveillance is another component of the sentinel surveillance system in Hong Kong. Specimens collected from sentinel locations were sent to the Public Health Services Laboratory for the purpose of surveillance. The clinicians who collected the specimen from the patient would not be notified of the result of laboratory testing. It is therefore less likely that the clinician would selectively choose patients for sample collection. The laboratory data reported by the Public Health Services Laboratory also include laboratory test results on inpatients admitted to local hospitals and occasional outpatients, and these are not distinguished in the data.

In this study, we used proxies representing virus activities of influenza virus and RSV. The proxy for weekly influenza activity was measured as the product of the weekly proportion of specimens collected in the sentinel surveillance system tested positive for a specific virus type/subtype and the weekly proportion of outpatient consultations due to influenza-like illness. In a previous study, we used data collected during the 2009 influenza pandemic and demonstrated that this proxy provides a good indication of incidence of H1N1pdm09 virus infections in the community (Wong *et al*. 2013 AJE). For RSV activity, we used the weekly proportion of specimens collected in the sentinel surveillance system tested positive for RSV as the proxy, which was suggested to be a better proxy in another study (E. Goldstein, pers. comm.).

### Statistical model

We examined influenza-attributable excess mortality associated with all-causes, cardiovascular diseases (ICD-9: 390-459; ICD-10: I00-I99) and respiratory diseases (ICD-9: 460-519; ICD-10: J00-J99). A linear regression model was used to estimate the association between the weekly death rates and all the covariates according to the following regression equation:

where *E*(*M*_*i*_) is the expected mortality rate in week *i*. β_1_–β_5_ denotes the effects on mortality associated with activities of different influenza virus subtypes and respiratory syncytial virus (RSV). sH1_*i*−1_, sH3_*i*−1_, *B*_*i*−1_ and pH1_*i*−1_ represent the weekly virus activity of seasonal influenza A(H1N1), A(H3N2) and B, and 2009 pandemic influenza A(H1N1), measured as the product of the weekly proportion of specimens (collected by the local surveillance system from hospitals and outpatient clinics) tested positive with the specific virus subtype and the weekly proportion of outpatient consultations due to influenza-like illness (ILI). The RSV activity RSV_*i*-1_ was measured as the weekly proportion of specimens tested positive for the virus. *P*_*i*−1_ is the pandemic term used in the model allowing for differences in the number of specimens collected for virus testing by the local surveillance system during the pandemic and non-pandemic period. The pandemic period was defined as the time period between the date when the Hong Kong government declared to include influenza A(H1N1pdm09) as statutorily notifiable disease on 27 April 2009 and the date when the influenza response level in Hong Kong was changed from ‘Emergency’ to ‘Alert’ on 24 May 2010. β_6_ and β_7_ are the effect of temperature and humidity on mortality, modelled as cubic smooth splines of the trends in the weekly temperature and absolute humidity *T*_*i*−1_ and *H*_*i*−1_, with the degree of freedom df_*T*_ and df_*H*_. β_8_ is the effect caused by changes in temporal trends on mortality. β_9_ refers to the effect caused by the transition in ICD coding system in Hong Kong, that is, from ICD-9 (1998–2000) to ICD-10 (2001–2011). ε_*i*_ is an error term, assumed to follow a normal distribution with constant variance over time. A 1-week lag was assumed between the time-varying covariates and mortality.

### Age-standardization of excess mortality risk

We derived the all-age excess mortality risk from the age-specific estimate of excess mortality by direct standardization using the following equation:

where *M*_*j*_ is the age-standardized excess mortality risk associated with a specific virus during the time period *j*. *m*_*i,j*_ represents the estimated excess mortality risk for the age group *i* in the time period *j*. The crude excess mortality risk for each age group was weighted by the age distribution of population size during April 2009 to March 2011, which covers the epidemic waves investigated in the study. *W*_*i*_ is the population weight for the age group *i* and measured by the following equation:

where  is the mean population size for the age group *i*, and  is the mean of the total population size during the time period defined for the study.
